# AI Model for Prostate Biopsies Predicts Cancer Survival

**DOI:** 10.3390/diagnostics12051031

**Published:** 2022-04-20

**Authors:** Kevin Sandeman, Sami Blom, Ville Koponen, Anniina Manninen, Juuso Juhila, Antti Rannikko, Tuomas Ropponen, Tuomas Mirtti

**Affiliations:** 1 Medicum and Research Program in Systems Oncology, Faculty of Medicine, University of Helsinki, P.O. Box 63, 00014 Helsinki, Finland; antti.rannikko@hus.fi (A.R.); tuomas.mirtti@helsinki.fi (T.M.); 2 Department of Pathology, Division of Laboratory Medicine, Skåne University Hospital, Jan Waldenström Gata 59, 20502 Malmö, Sweden; 3 Aiforia Technologies Plc., Tukholmankatu 8, 00290 Helsinki, Finland; sami.blom@aiforia.com (S.B.); ville.koponen@siili.com (V.K.); anniina.manninen@aiforia.com (A.M.); juuso.juhila@aiforia.com (J.J.); tuomas.ropponen@aiforia.com (T.R.); 4 Department of Urology, Helsinki University Hospital, P.O. Box 340, 00029 Helsinki, Finland; 5 Department of Pathology, HUSLAB Laboratory Services, Helsinki University Hospital, P.O. Box 720, 00029 Helsinki, Finland

**Keywords:** prostate cancer, biopsy, radical prostatectomy, grade group, artificial intelligence, deep learning

## Abstract

An artificial intelligence (AI) algorithm for prostate cancer detection and grading was developed for clinical diagnostics on biopsies. The study cohort included 4221 scanned slides from 872 biopsy sessions at the HUS Helsinki University Hospital during 2016–2017 and a subcohort of 126 patients treated by robot-assisted radical prostatectomy (RALP) during 2016–2019. In the validation cohort (n = 391), the model detected cancer with a sensitivity of 98% and specificity of 98% (weighted kappa 0.96 compared with the pathologist’s diagnosis). Algorithm-based detection of the grade area recapitulated the pathologist’s grade group. The area of AI-detected cancer was associated with extra-prostatic extension (G5 OR: 48.52; 95% CI 1.11–8.33), seminal vesicle invasion (cribriform G4 OR: 2.46; 95% CI 0.15–1.7; G5 OR: 5.58; 95% CI 0.45–3.42), and lymph node involvement (cribriform G4 OR: 2.66; 95% CI 0.2–1.8; G5 OR: 4.09; 95% CI 0.22–3). Algorithm-detected grade group 3–5 prostate cancer depicted increased risk for biochemical recurrence compared with grade groups 1–2 (HR: 5.91; 95% CI 1.96–17.83). This study showed that a deep learning model not only can find and grade prostate cancer on biopsies comparably with pathologists but also can predict adverse staging and probability for recurrence after surgical treatment.

## 1. Introduction

Tissue morphology in digital images can be assessed as accurately and efficiently as with conventional light microscopy [1,2]. Pattern recognition methods are available for conventional morphological staining and immunohistochemical antigen detection, which adds significant diagnostic value in clinical pathology [3,4]. More developed, machine-learning-based methods have been successfully used to stratify inter alia lung cancers that harbor different mutational statuses and also gastrointestinal cancers based on microsatellite instability status [5,6].

There is an unmet need to improve cancer outcome prediction based on histological cancer samples. AI methods are applicable for the recognition of cell and tissue components (epithelium, stroma, vessels, immune cells, etc.) in addition to their distribution and proportions. These methods have been successfully used to predict the progression of cancer [7,8]. Computer vision and neural networks have also been used in the search for predictive features that are independent of the traditional parameters, such as stage and grade [9,10,11]. There is growing interest toward prostate cancer (PCa) histological grading by means of neural networks, and some recent studies have managed to touch upon the possible use of deep learning (DL) methods in the prediction of PCa progression and survival [12,13,14]. AI, or more precisely trained neural networks, has shown great promise in detecting histological morphologies and changes that pathologists have traditionally determined [15]. Gleason grading is the strongest predictor of survival after primary therapy for PCa. From the introduction of the Gleason Score (GS) in 1966 until now, Gleason grade patterns have been modified and recapitulated into GS 6 to 10. A new system with five distinct grade groups (GGs) has been adopted for use in conjunction with the Gleason grading, as it is simpler and reflects more correctly the PCa outcome [16]. However, as GGs are based on the modified GS groups, the accuracy of Gleason grading is as important as before (Table 1) [16].

In terms of survival, there is clear evidence [17,18,19] of the difference between GG2 and GG3, which emphasizes the importance of accurate and standardized assignment of Gleason grading patterns. The interobserver reproducibility of grading among urologic pathologists has been shown to be within an acceptable range [20]. However, among general pathologists, there is still a need for training to reduce the interobserver variability in Gleason scoring [21]. The cribriform growth pattern of the Gleason grade 4 has a high level of reproducibility between pathologists and is a highly specific predictor for metastasis, disease-specific death, and biochemical recurrence (BCR) [22,23,24]. The independent predictive value of high Gleason grade patterns 4 and 5 has been previously shown as well [25,26].

PCa-related intra- and interobserver variation has recently been challenged by AI methodology. Specifically, neural networks have mostly succeeded in binomial classification of benign tissue vs. cancer or reached the level of expert pathologists in grading prostate biopsies [12,13,14,27]. To become usable in everyday pathology practice and decision making, any neural-network-based algorithm must therefore succeed in outcome prediction. Thus far, only a few studies have tried to address this problem in PCa, and some of these studies have shown no less than human-level results when performed in radical prostatectomy (RALP) cohorts [12,14].

For a long time, training new AI-augmented image analysis models have been labor intensive and required expert annotations of large data sets. Recent advances in neural networks and other AI models have enabled faster and more efficient development of detection of changes in histological tissue slides.

Here, we have trained a robust Gleason grade classifier in prostate biopsies by using a relatively small training cohort. We have endeavored to create an efficient and accurate algorithm that also reproduces the characteristics of grading for the prediction of cancer recurrence after primary therapy.

## 2. Materials and Methods

Our cohort consisted of 750 patients undergoing prostate biopsy at the Helsinki University Hospital (HUS) between 2016 and 2017. For cohort demographics, see Table 2.

**Figure 1:**
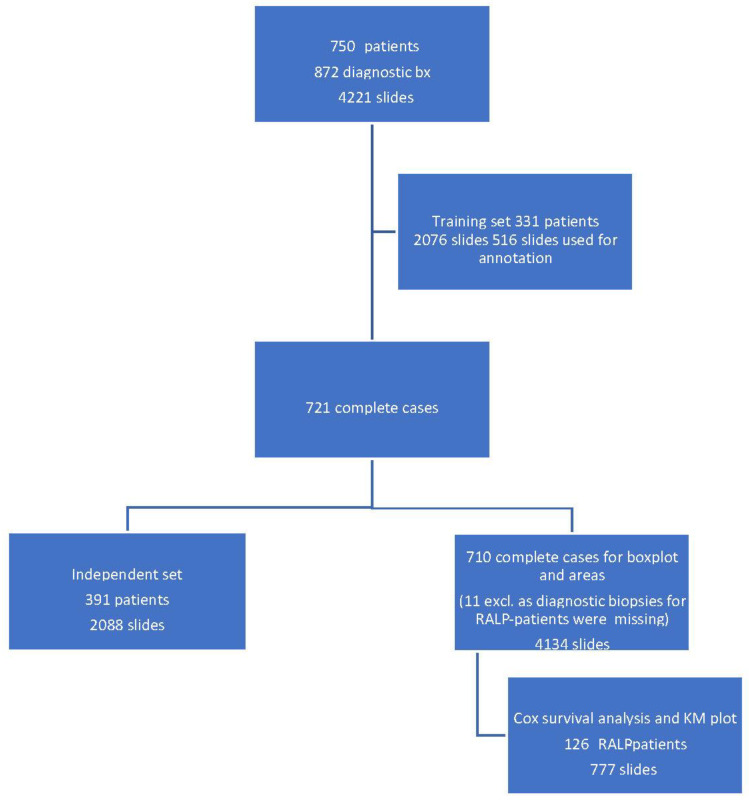
Workflow of the study cohort data selection depicting a training set, an independent set, and radical prostatectomy (RALP) patient cohort from 872 prostate core biopsy series.

A total of 872 biopsy sessions resulted in 4221 hematoxylin-and-eosin-stained (H&E) slides that were scanned for AI development (Figure 1).

Clinical and pathological data were collected for all the patients. For RALP patients, information about extra prostatic extension (EPE), seminal vesicle invasion (SVI), node status (NS), and pathologic stage (pT) was collected from electronic surgical pathology reports. Adverse pathologic findings in RALP specimens were defined as EPE, SVI, or positive NS. All post-operative prostate-specific antigen (PSA) values during the follow-up were collected for survival analysis. Detection of two consecutive post-operative PSA values equal to or higher than 0.2 ng/mL was considered as a BCR with the date of the first PSA value as the date of the event. The follow-up of the RALP cohort ended in May 2020. Of the biopsies, 79% were diagnosed by nine pathologists who sign out prostate biopsies at least on a weekly basis and the remaining 21% were diagnosed by six pathologists with uropathology experience less than that. All the 15 pathologists were considered equal in the analysis.

The H&E biopsy slides were scanned using a Pannoramic 250 Flash III scanner (3DHistech, Budapest, Hungary) with a 20× objective (numeric aperture 0.8) at 0.26 µm/pixel resolution, resulting in an optical magnification of 41×. The digital slide images were compressed to 50% in JPEG format and uploaded to Aiforia software (Aiforia Technologies Plc., Helsinki, Finland) for annotation, development, and testing of the AI algorithm.

For the AI algorithm, two independent convolutional neural networks (CNNs) were trained for multi-class semantic segmentation of tissue (CNN-T) and Gleason grades (CNN-GG). The training was fully supervised, and the training data were annotated as strong pixel-level multi-class segments for both tissue and Gleason grades in the Aiforia software by two expert uropathologists (K.S. and T.M.) in consensus. All training data were digitally augmented for geometric (i.e., rotation and scale) and photometric (i.e., contrast and noise) transformations to avoid overfitting and to ensure generalization of the CNNs. CNN-T and CNN-GG included 12 convolutional layers each and were trained using a 90 µm × 90 µm and a 100 µm × 100 µm field of view. These were used for multi-class logistic regression loss function. The final AI algorithm inference pipeline combined both CNN-T and CNN-GG in a nested design where Gleason grades were detected within the detected tissue regions.

To enable comparison between AI model and original clinical pathological diagnoses, each determined by 1 of the 15 pathologists, the proportions of each growth pattern, i.e., Gleason grade 3, grade 4, grade 4 with cribriform pattern, and grade 5, in each set of digital diagnostic biopsies were calculated to generate a Gleason score. A GS is based on the reporting criteria for prostate biopsies detailed in the International Society of Urological Pathology (ISUP) Consensus Conference in 2014, which addressed the most common and the highest Gleason grade patterns [16]. The result was then converted to an ISUP grade group (Table 1) to enable cross-tabulation and comparison between AI and pathologists for GG in a 6-tier-grouping (benign and GGs 1–5).

**Figure 2:**
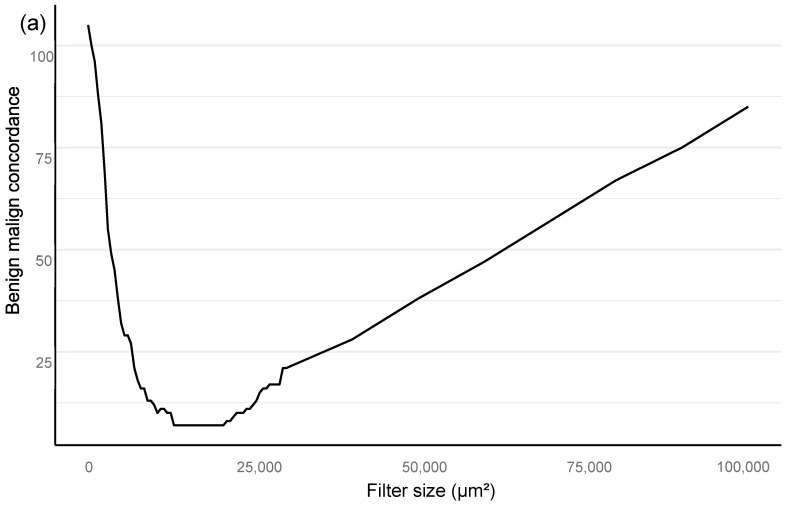

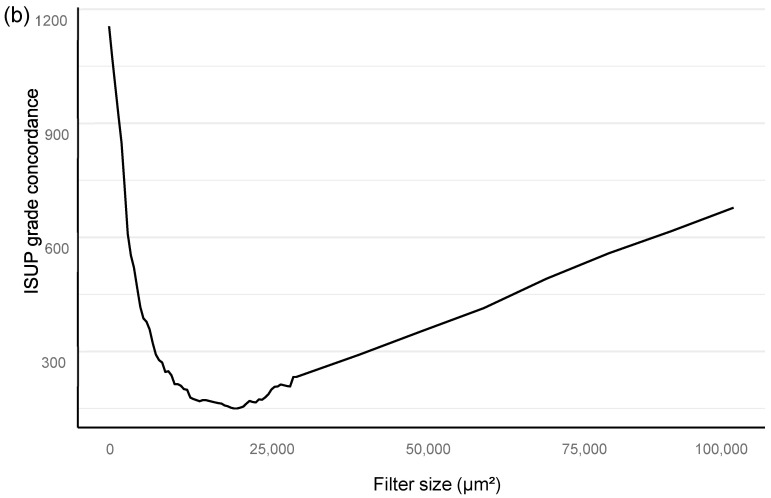
Bloom filter for the determination of the minimal filter size for benign versus malign tissue (**a**) and grade group difference (**b**) based upon clinical performance of the algorithm compared to clinical diagnosis.

Training of the algorithm was performed as iterations to optimize the performance of the AI algorithm. The training included primary annotations, correction of annotations, and addition of new sections from independent material that were based upon outliers in the AI vs. the pathologist confusion matrix. An area filter curve that was based upon this confusion matrix was then constructed to find the optimal minimum for the filter area, which corresponded with benign vs. malignant differentiation and also GG stratification (Figure 2; see also the Section 2.1).

In the algorithm training phase, the clinically applicable filter size was determined iteratively based on urological pathology experts.

The filter curve was configured to be compliant with a clinical workflow. The AI algorithm training material consisted of a subset of 516 slides selected from 2076 slides that came from 331 patients. A total of 3606 separate glandular areas and stromal background were annotated on the training set slides, which resulted in a total area of 94 mm^2^ of completely annotated prostate tissue (Table 3).

The independent control set comprised 2088 slides taken from 391 patients. Figure 1 shows the full study flowchart with patient stratification. The median number of slides in a complete preoperative slide set per patient (total number of patients 721) was 6, and most of the slides represented systematic biopsies (2 cores per slide). The average area of tissue was 25 mm^2^ per slide, consisting of two needle biopsies and two levels each, which then equals approximately 6.3 mm^2^ per individual needle core level on the slide. Therefore, the area of the annotated training data corresponded to a total area of four slides or 15 needle cores.

The RALP-specimen-related-adverse-outcome data and the follow-up PSA data of the RALP subcohort are completely independent from AI training and the associated data. Thus, the survival analysis was executed blinded to the annotating pathologists, and post-operative data were not used in the generation of the AI algorithm. The data were accessed and collected between October 2016 and May 2020 from the electronic health records at the HUS Helsinki University Hospital. The original data were accessed by using the patients’ social security numbers, but in the study database, all data were pseudonymized according to the study approval by the Helsinki University Hospital (HUS) (latest update 16 November 2019, diary number §166 HUS/44/2019). As this was a retrospective registry study, no informed consent was required as stipulated by Finnish national and European Union legislation. The study was conducted according to the Declaration of Helsinki.

### 2.1. Statistical Analyses

All statistical analyses are assessed for a filter size of 15,000 square microns, which showed the best performance in the Bloom filter analysis for the best determinant of class stratification (Figure 2). ROC AUC analysis was performed and predictive accuracies determined for the algorithm. Cohen’s weighted kappa coefficient was used to measure interrater agreement between the AI model and each of the original classifications. Generalized linear models were applied to determine the relationship between the clinical GG and the AI-determined tumor area. Multivariable logistic regression models were used to determine the relationship between biopsy AI results and adverse outcomes on the RALP specimen (EPE, SVI, or positive NS). Cox proportional hazard regression models and Kaplan–Meier survival curves were applied to assess the predictive value of the AI-based GG and the original GG on BCR during the post-operative follow-up. An alpha level of 0.05 was used for statistical significance. All statistical analyses were made using R Statistical Software version 4.1.0 [28] and using the caret and survival package [29,30].

## 3. Results

### 3.1. Artificial Intelligence Algorithm Performance and Accuracy

**Figure 3:**
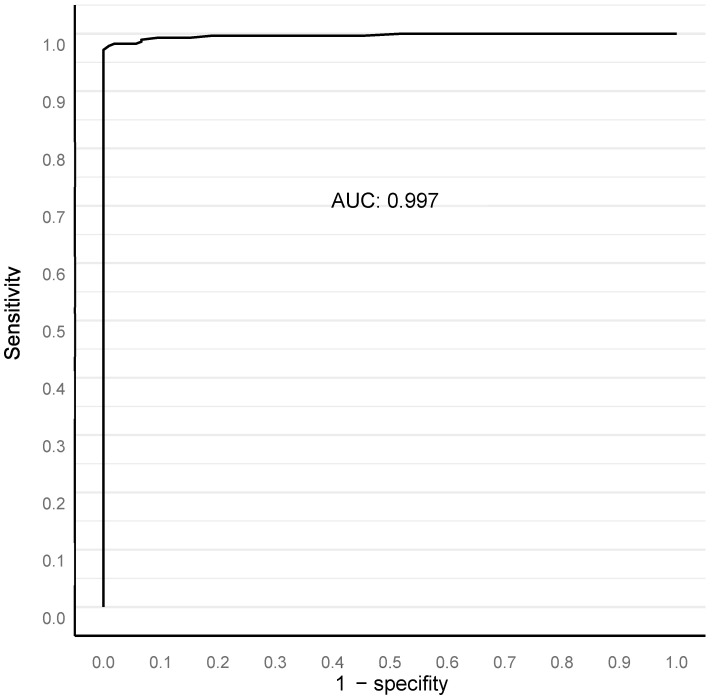
ROC AUC analysis for benign versus malignant compared to clinical diagnosis at full biopsy session level, with a 15,000 square micron filter applied (see text).

As our approach in finding the best deep neural network solution is based on pixel-level annotations and full biopsy session (patient-level) ground truth for a GG, we mathematically modeled a minimum area for histological class detection that would not miss a clinically significant cancer. The Bloom filter curve method determined the best filter size for benign vs. malignant and GG classification, based upon a comparison of the sensitivity and specificity of the AI model to the pathological report (Figure 2). This analysis was independent of the outcome validation data. The filter area is defined as the minimal solitary area in square micrometers accepted as a relevant finding on a single-slide level. The optimal filter area for our algorithm turned out to be at 15,000 square μm (Figure 2), resulting in an AUC 0.997 in ROC AUC analysis for the detection of malignant vs. benign (Figure 3).

**Figure 4:**
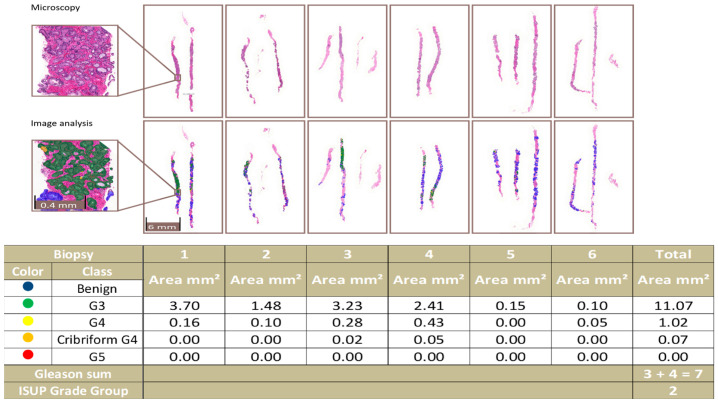
An example of a complete biopsy session slide set. Image analysis data are produced by pixel-level multi-class semantic segmentation at the level of the individual biopsy slide and compiled at the level of the complete slide set. In the example, the patient-level ISUP grade group is 2.

Even from a clinical perspective, the filter still includes small tumor areas recognizable by a uropathologist in biopsy series, as illustrated in Figure 4.

We optimized the algorithm by comparing the results of AI and the pathologist at original diagnosis in a confusion matrix. The chosen algorithm and filter size resulted in a confusion matrix with complete agreement on a patient level in 260 (66%) of 391 independent biopsy sessions. AI diagnosed five false negative results (1%) and two false positives (both originally benign to GG1). Disagreement by one GG in PCa between AI and the pathologists was seen in 111 cases (24%) (Table 4).

Sensitivity, specificity, negative predictive value (NPV), and positive predictive value (PPV) were calculated for the benign class and also for each GG (Table 5).

The overall weighted kappa value obtained by the algorithm was 0.76 (*p* < 0.0001). Predictive values for the algorithm performance were determined for clinically relevant cohorts ISUP GG1−2 versus ISUP GG3 to 5 (Table 6).

### 3.2. Determinants of Cancer Extent by Image Analysis Correlated with Clinical Diagnosis

**Figure 5:**
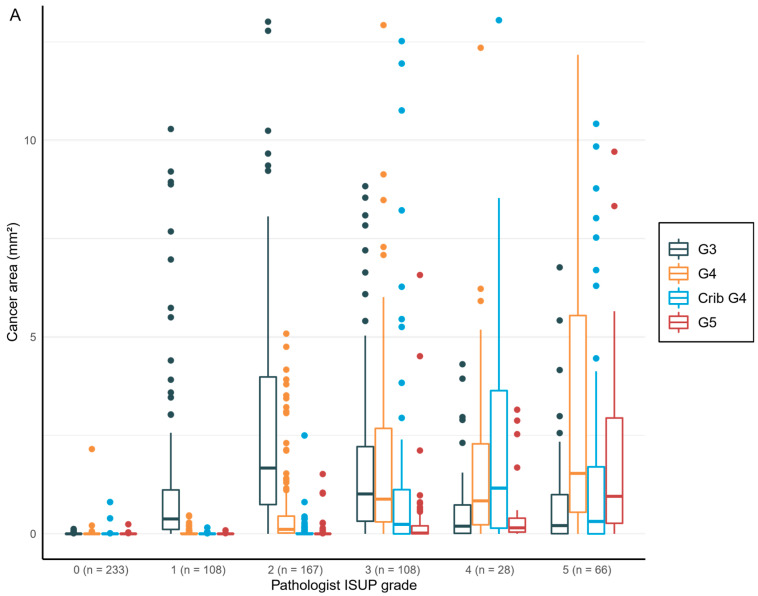

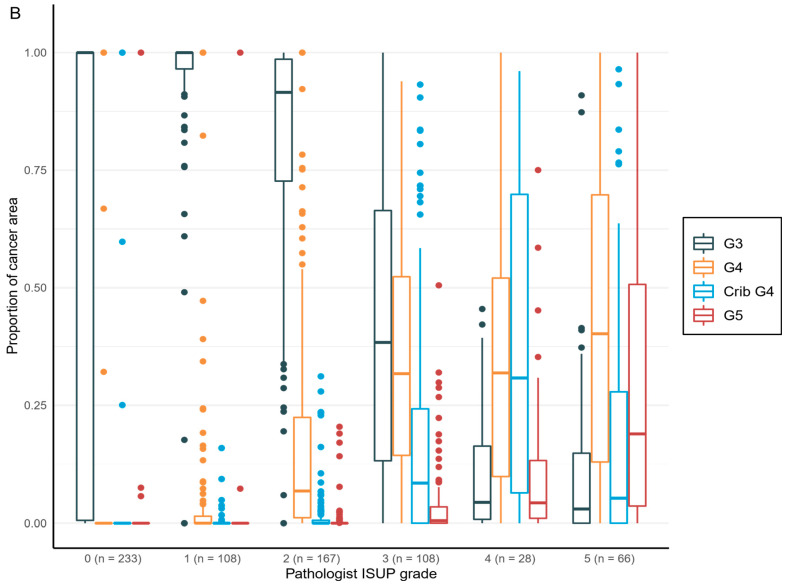
Distribution of the Gleason grade in area (**A**) and percentage of the cancer area (**B**) by AI compared to the grade group by the pathologist. Total n = 710.

The total area and the proportion of cancer detected by AI per biopsy session were plotted against the pathologist’s diagnosis of GGs and showed a logical shift of AI-diagnosed grade patterns that correlated with the pathologist’s diagnoses (Figure 5).

Logistic regression models complemented the distributions shown in the boxplots with statistically significant relationships between the tumor area graded for all classes by AI and the clinical diagnosis according to the pathologists (Table 7).

### 3.3. Artificial Intelligence Predicted Biopsy-Adverse Pathological Findings after Surgical Treatment

AI comparisons between the biopsy session GG and the RALP GG were down- and upgraded in both the AI-determined biopsy GG and the human-observer-determined biopsy GG. In the confusion matrixes, the deviation from the mid was found to be larger in the AI-graded biopsy than in the original biopsy (Table 8). The difference is logically explained by the higher sensitivity of the AI algorithm in detecting grade patterns 4 and 5 (Table 4), which is especially well illustrated in RALP GG3. Moreover, the higher number of GG5 in AI-determined biopsy grades compared with pathologists’ GG5 (Table 8) was associated with the reported tertiary grade pattern in RALP (in 16/26 of the GG3–5 patients). The tertiary pattern as defined in the Gleason grading system for the RALP specimen was not included in the GS of these patients.

**Figure 6:**
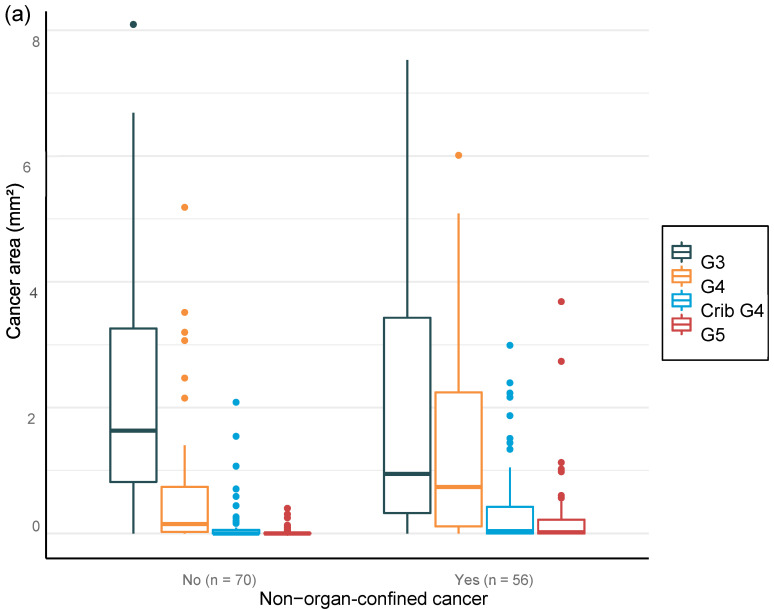

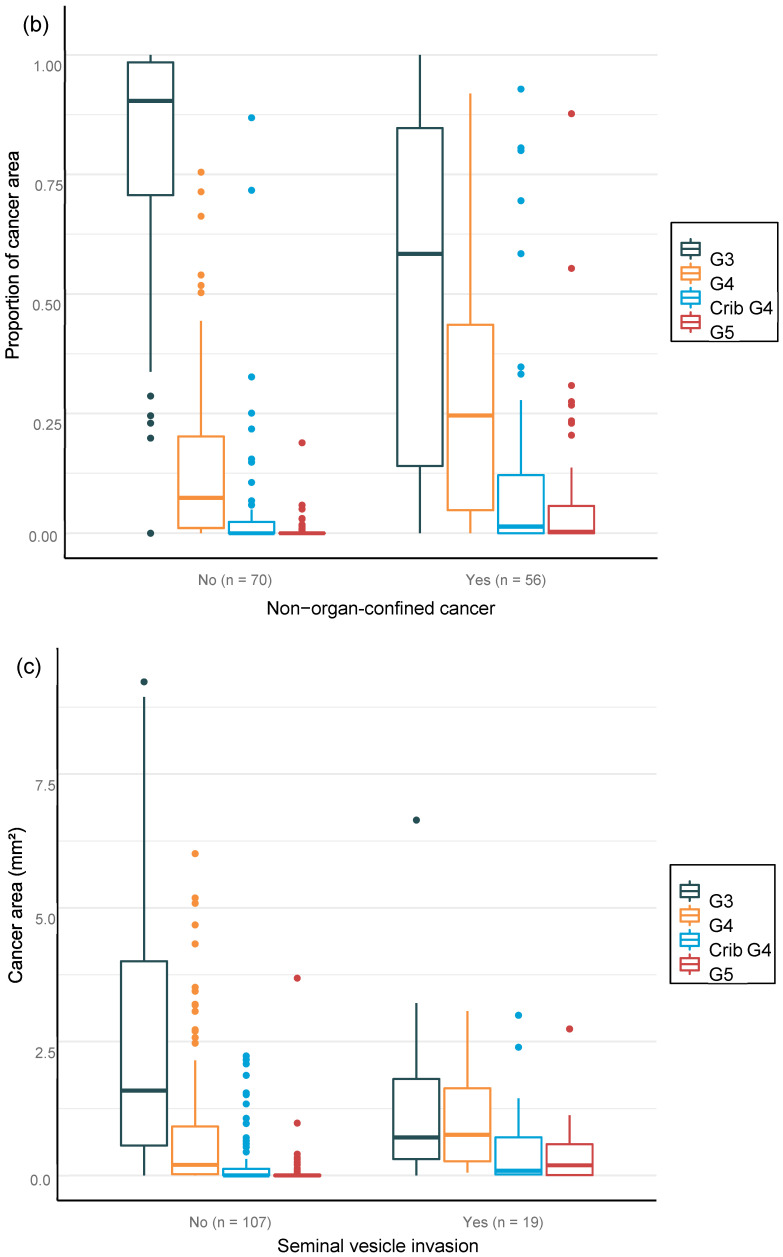

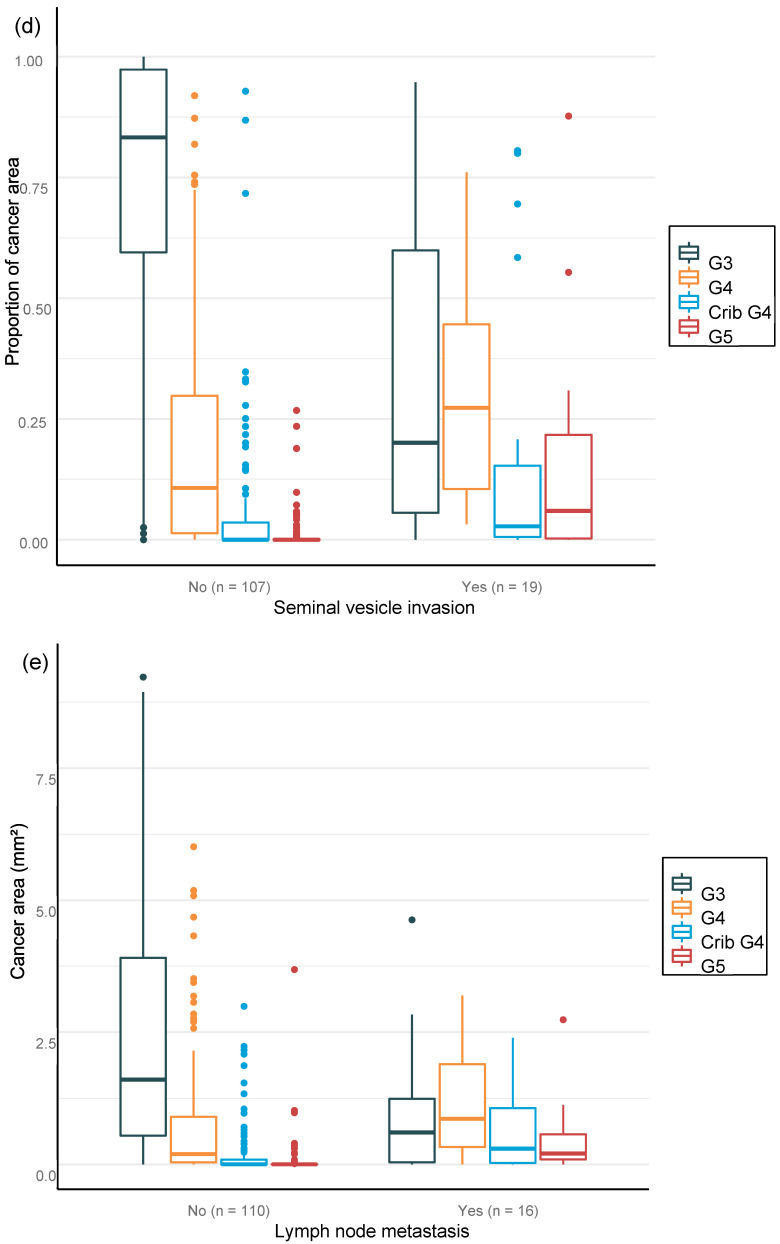

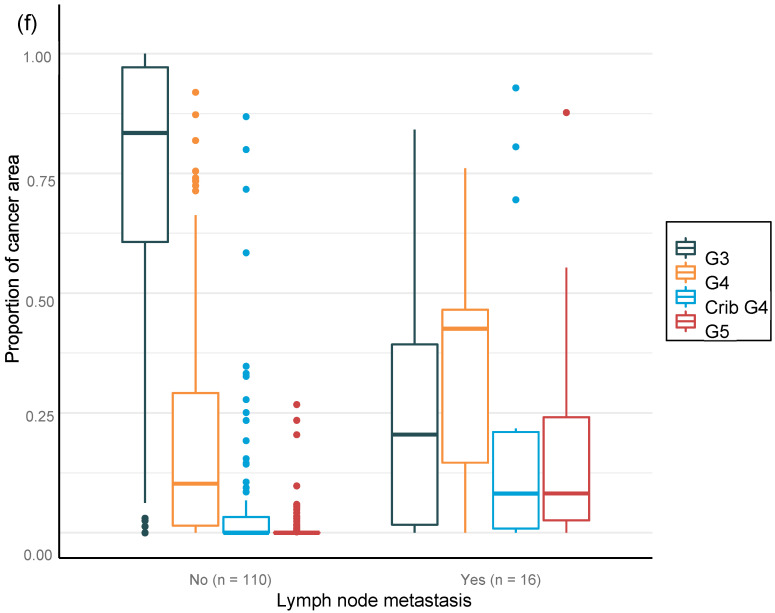
Distribution of Gleason grade in area (**a**,**c**,**e**) and percentage of cancer area (**b**,**d**,**f**) by AI on biopsy session compared to adverse events non-organ confined (**a**,**b**), seminal vesicle invasion (**c**,**d**), and lymph node involvement (**e**,**f**) at prostatectomy.

The value of AI in predicting adverse cancer extent after surgery was analyzed for the total area. Moreover, the proportion of cancer detected by AI per biopsy session was plotted against the adverse pathological findings at prostatectomy (Figure 6).

Multiple logistic regression analysis of the area of grade pattern 5 especially showed statistically significant correlation with adverse pathological findings. The AI-assigned areas of cribriform grade 4 and grade 5 in biopsies correlated significantly with SVI and LN metastasis at RALP (Table 9).

### 3.4. Neural-Network-Based GG Was Non-Inferior to the Human Observer in Predicting Disease Recurrence

**Figure 7:**
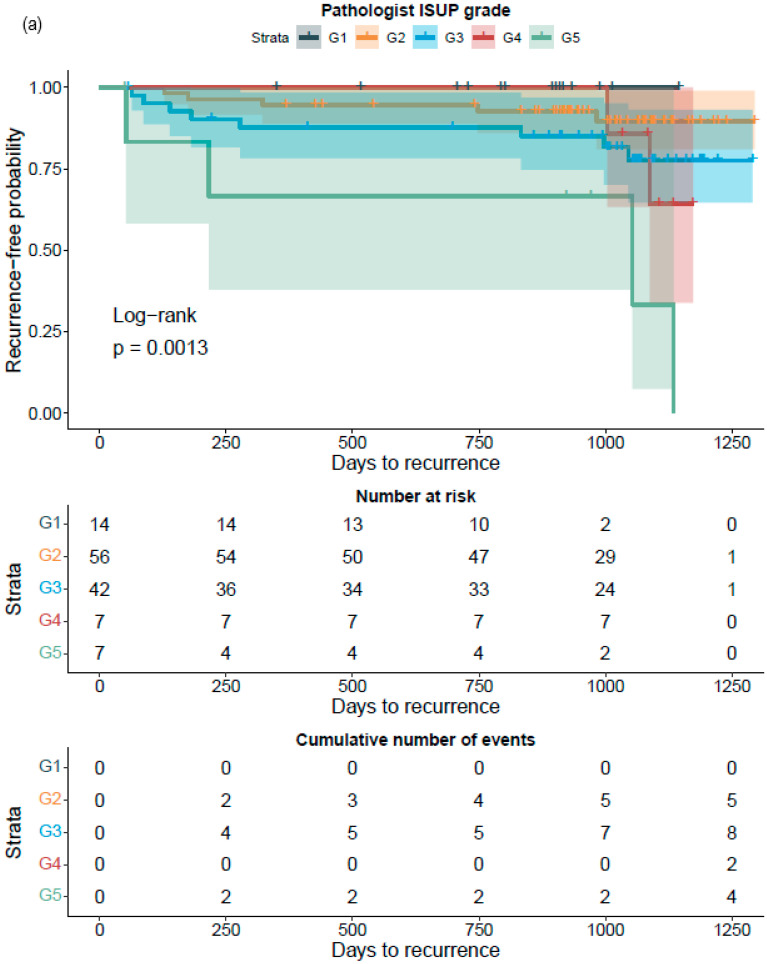

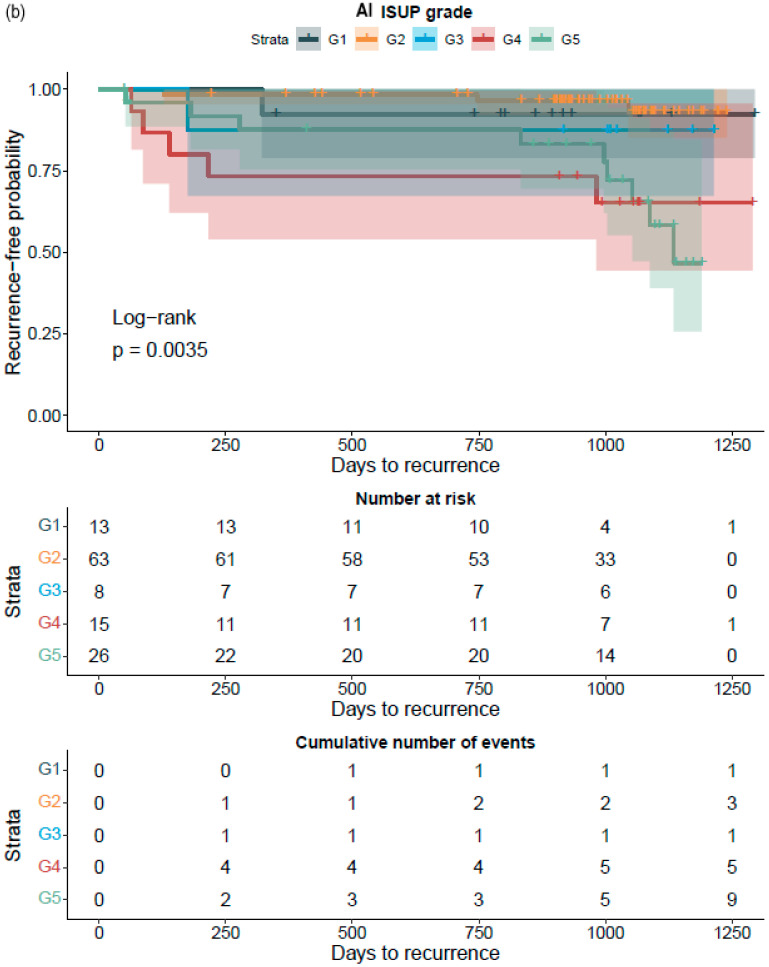

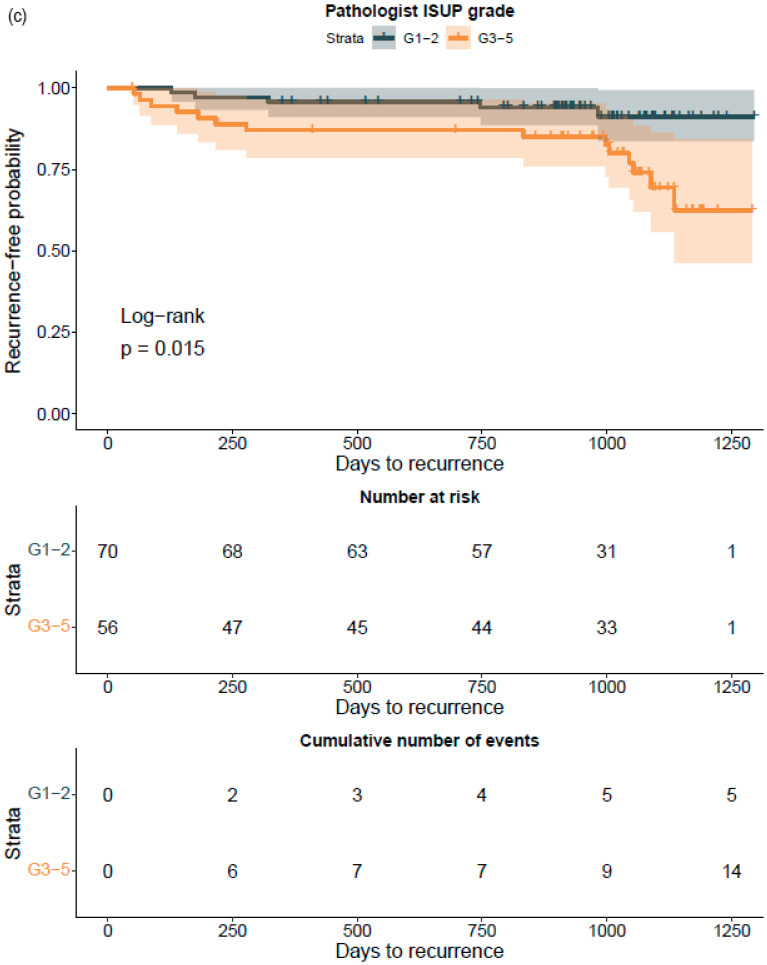

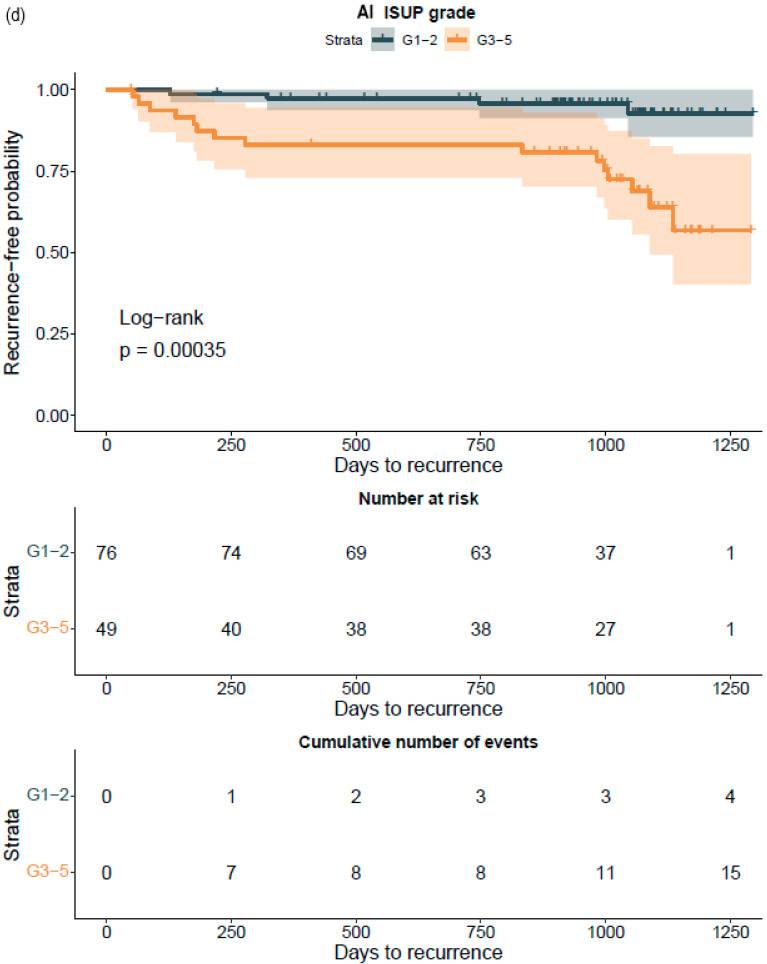
Kaplan–Meier survival analysis for biochemical recurrence on non-stratified (**a**,**b**) and stratified (1–2 versus 3–5) (**c**,**d**) cancer-positive biopsies for the prostatectomy cohort. The graph shows 95% confidence intervals.

Kaplan–Meier survival analysis showed that the GG assessed either by the pathologists or by AI significantly divided the patients into groups with different probabilities for BCR after RALP. This was also true after the stratification of patients into groups of GG1–2 and GG3–5, where the separation of the survival curves was more evident for the neural-network-based GG assessment (Figure 7).

A Cox proportional HR analysis for BCR revealed that an increase in the GG was also associated with an increased risk for BCR irrespective of the observer, i.e., pathologist or AI (Table 10). This was also the case after dichotomizing the cohorts into GG1–2 and GG3–5. A proportional increase in grade pattern 3 was associated with a decreased risk for BCR. Conversely, an increase in the proportion of grade pattern 5 was significantly associated with an increased risk for BCR (Table 10).

## 4. Discussion

To the best of our knowledge, this is the first report that shows the prognostic value for a CNN-model-based grade group assignment in diagnostic prostate biopsies. Not only did the model accurately classify the diagnostic biopsies, it also accurately categorized patients to different risk groups for BCR after surgical treatment. Although our RALP cohort is limited in size, and only a limited number of biochemical relapses were observed due to short follow-up times, the strength of the AI model is shown in the predictive accuracy, which is on a par with the pathologist, ad minimum.

In PCa diagnostics, the correct identification of benign tissue is not an issue generally. Furthermore, this task can be performed by an AI-based approach, as shown by Campanella et al., who obtained slides from multiple institutions to develop an algorithm for cancer detection [27]. However, their algorithm remains a binary cancer detection algorithm and its ability to predict the risk of disease recurrence has, to the best of our knowledge, not been reported. However, the current clinical challenges relate to consistently accurate grading of prostate biopsy samples to support clinical decision making.

Compared to other similar, although single-slide-based studies, our undoubtedly more clinically oriented study is unique, as it used a neural network algorithm that reached the performance of pathologists at the patient level in the whole biopsy session [12,13,14]. The practical clinical setting, with 15 independent pathologists with varying experience in prostate diagnostics who signed out the biopsies, did not compromise our results. The algorithm was trained by two uropathologists, who annotated strong image labels, which resulted in a small training set compared with the independent testing group. The strong image labeling seemed to improve the results for differentiating GG1–2 versus GG3–5, compared with a previous report that used pure Gleason 3 + 3, 4 + 4, and 5 + 5 slides for training its algorithm [13]. Pure tumor differentiation that just represents one GG is seldom observed in clinical practice as it is a rare phenomenon in biology.

The glandular architecture-based GG has been the most significant prognostic and predictive factor since its implementation in the 1960s and its subsequent gradual adoption [31,32]. The growth pattern assessment is forced into scale-wise grades, which undoubtfully reduces the meaningful information inherent to gland formation and cellular morphology. Modification of the Gleason grading system over the years has led to a regression toward the mean, i.e., clumping of GSs into intermediate scores of 7 and 8. The newly adopted grade grouping system in practice reproduces the same problem [16]. There have been efforts to quantify and weigh the significance of growth patterns with the means of visual quantification of different growth patterns [32] in addition to computer-vision-based algorithms [14]. In our study, the notion is that the “general pathologist”-determined diagnostic GG tended to underestimate the presence of Gleason 4 or higher-grade patterns, as the expert pathologist-trained AI algorithm detected smaller, but clinically significant, foci of high-grade cancer. In the future, more consistent recognition of growth patterns, their proportions, and their relationships aided by computer vision and AI models will provide more stratification into the rigid GG categories and aid in personalized treatment approaches. AI-based grading creates consistency and reduces interobserver variation, which is characteristic of the categorical grouping performed by pathologists. A trained algorithm would not perform differently from day-to-day, as a human observer does. However, at the institutional level, there might be issues related to differences in the adopted interpretation of grade patterns. This creates a challenge for the generalization of a new grading algorithm. This problem may, however, be overcome by training the algorithm in-house with locally selected cases. Our algorithm also has real potential to help those pathologists who are not genitourinary oriented or have limited experience in prostate pathology.

We were able to confirm that the AI-based area of cribriform G4 and G5 in prostate biopsies correlated with subsequent RALP adverse outcomes such as seminal vesicle invasion and positive lymph node status. The area of G5 in the biopsies significantly correlated with non-organ-confined disease. We observed that the proportion of GG pattern 5 is a strong predictor of BCR. This is in line with previous publications that show that the presence of any GG 5 pattern predicts an elevated probability of a relapse after primary treatment [26,33]. This is the first study to focus on patient-level outcome after prostatectomy based upon AI grading of full series of preoperative diagnostic prostate biopsies per patient. Our results confirm, ad minimum, a predictive performance compared to that of a human observer and suggest refined predictive accuracy that should subsequently be confirmed after a longer follow-up period. We chose mathematical modeling to work with a 15 000-micron filter to reproduce a comprehensive clinical workflow. The algorithm did not miss any cancer areas, although the use of the filter as an aid for fluent clinical workflow produced five false negative findings, of which three represented GG1, one represented GG2, and one GG3. Only two patients with benign biopsies were falsely classified as cancerous, and both had GG1. There is a definite need to develop more advanced AI models to detect small Gleason grade pattern 3 and 4 areas accurately if the sole aim is to mimic human ground truth with known inherent variability.

A weakness of the study is the lack of an external-site validation cohort. Similar, well-curated cohorts with matched preoperative biopsies, surgical specimens, and follow up for BCR are scarce. Moreover, there are differences in the biopsy techniques and tissue preparation methods between institutions. It is known that the performance of an algorithm usually does not reproduce as accurately in external validation cohorts as in the original cohort. All these limitations underscore the need for future prospective, preferably randomized, clinical trials to show that computer-vision-based cancer detection and grading performs similarly to the current gold standard of the expert human eye [15,34].

Ultimately, histology may include biologically and clinically relevant signals currently overlooked by the human eye, which may improve our ability to predict cancer behavior [35]. The AI-based methods may help to stratify patients with different life expectancies within a given GG. The future of clinical decision making is likely shared decision making conjoined with supporting multidisciplinary prognostics models. For example, it has been shown in brain tumors that an AI algorithm complemented with molecular characteristics provides better predictive information than the information about the morphological differentiation or mutational status alone [36]. DL models have also been used to explore molecular determinants of treatment response in PCa [37]. It remains to be shown whether the biological signals and drivers of PCa progression and treatment resistance can be predicted in the tissue phenotype at a level that is clinically applicable [38,39].

## 5. Conclusions

This study shows that AI can find and grade PCa on biopsies comparably with pathologists. AI predicts adverse staging in a prostatectomy specimen and probability for post-operative BCR equally to a pathologist.

## Figures and Tables

**Table 1:** Grade grouping based upon Gleason scores.

ISUP Grade Group	Gleason Score
GG1	3 + 3
GG2	3 + 4
GG3	4 + 3
GG4	4 + 4, 3 + 5, 5 + 3
GG5	4 + 5, 5 + 4, 5 + 5

ISUP = International Society of Urological Pathology.

**Table 2:** Characteristics of the men diagnosed with prostate cancer and the subcohort that underwent surgical treatment.

Full Cohort		n, Mean, or Median	Percentage, SD, or IQR
Patients, n		750	100
Age (years), mean (SD)		68.1	8.09
Biopsies, n		871	100
Biopsy series, n (%)	1	653	87.07
	2	84	11.2
	3	3	0.4
	4	9	1.2
	5	0	0
	6	1	0.13
Biopsy ISUP grade group, n (%)	0	258	34.4
	1	113	15.07
	2	169	22.53
	3	114	15.2
	4	28	3.73
	5	68	9.07
PSA (ng/L), median (IQR)		8.5	4.5
**RALP Cohort**		**n, Mean, or Median**	**Percentage, SD, or IQR**
Patients, n (%)		126	100
Biopsies, n (%)		146	100
Age at RALP (years), mean (SD)		64.8	7.1
Biopsy series, n (%)	1	106	84
	2	20	16
Biopsy ISUP grade group, n (%)	0	0	0
	1	14	11
	2	56	44.44
	3	42	33
	4	7	6
	5	7	6
Preoperative PSA (ng/L), median (IQR)		8.5	4.5
Prostatectomy pT, n (%)	2	70	56
	3a	37	29
	3b	19	15
pN, n (%)	0	110	87
	1	16	13
BCR relapse PSA (ng/L), median (IQR) (n = 19)		0.59	1.07
Months to BCR relapse, median (IQR)		10.58	27.71

ISUP = International Society of Urological Pathology; PSA = prostate-specific antigen; RALP = robot-assisted radical prostatectomy; BCR = biochemical recurrence; pT = primary tumor pathological stage; pN = lymph node stage.

**Table 3:** Total area per annotated class in the training set of 516 biopsy slides.

Class	Total Area in mm^2^
Benign	58.24
G3	9.41
G4	10.27
G4 cribriform	6.28
G5	9.7

**Table 4:** Confusion matrix for the grade group in complete biopsy sessions by the pathologist versus AI. Distribution of patient-level grade groups (**A**). Percentage distribution (**B**).

**(A)**							
**Grade Group**		**Pathologist (k = 0.766)**					
		**0**	**1**	**2**	**3**	**4**	**5**
**AI**	0	104	3	1	1	NA	NA
	1	2	61	19	2	NA	NA
	2	NA	19	58	21	NA	1
	3	NA	1	2	10	2	1
	4	NA	1	3	12	2	4
	5	NA	NA	NA	22	14	25
**(B)**							
**Grade Group**		**Pathologist (k = 0.766)**					
		**0**	**1**	**2**	**3**	**4**	**5**
**AI**	0	95%	3%	1%	1%	NA	NA
	1	2%	73%	23%	2%	NA	NA
	2	NA	19%	59%	21%	NA	1%
	3	NA	6%	13%	63%	13%	6%
	4	NA	5%	14%	55%	9%	18%
	5	NA	NA	NA	36%	23%	41%

The blue shading represents the number (A) or percentage (B) of cases in each cell of the matrix.

**Table 5:** Accuracy of the AI algorithm: Predictive values for all classes.

	Class					
	Benign	GG1	GG2	GG3	GG4	GG5
**Cases, n (%)**	112 (28%)	85 (21%)	83 (21%)	68 (17%)	18 (5%)	31 (8%)
**Sensitivity**	0.98	0.72	0.70	0.15	0.11	0.81
**Specificity**	0.98	0.93	0.87	0.98	0.95	0.90
**PPV**	0.96	0.73	0.59	0.63	0.09	0.41
**NPV**	0.99	0.92	0.92	0.85	0.96	0.98

Accuracy = 0.67. Cohen’s weighted kappa = 0.77. *p* < 0.0001.

**Table 6:** Accuracy of the AI algorithm: Predictive values for dichotomized groups.

	Class				
	Benign versus Malign	Benign to Grade 1 versus 2–5	Benign to Grade 2 versus 3–5	Only Malignant Cases Grade 1 versus 2–5	Only Malignant Cases Grade 1–2 versus 3–5
**Number of Cases**	112/285	197/200	280/117	85/200	168/117
**Sensitivity**	0.98	0.89	0.98	0.75	0.96
**Specificity**	0.98	0.89	0.79	0.89	0.79
**PPV**	0.96	0.88	0.92	0.74	0.87
**NPV**	0.99	0.89	0.93	0.89	0.93
**Accuracy**	0.98	0.89	0.92	0.85	0.89
**Cohen’s Kappa (*p* < 0.0001)**	0.96	0.78	0.80	0.63	0.76

**Table 7:** Generalized linear modeling demonstrates a statistically significant relationship between the clinical GG and the AI-determined tumor area for each class for the independent set, indicating that every AI-determined-class-dependent tumor area attributes to the AI-assessed GG in a similar way as the pathologist.

Tumor Area in mm^2^	Odds Ratio	95% CI	*p*
G3	1.14	(1.08–1.19)	<0.0001 *
G4	1.14	(1.1–1.18)	<0.0001 *
Cribriform G4	1.22	(1.16–1.28)	<0.0001 *
G5	1.08	(1.05–1.12)	<0.0001 *

n = 397; *: *p*-values < 0.05 were considered significant.

**Table 8:** Cross tabulation showing down- and upgrading of the biopsy session GG in the RALP specimen.

	RALP GG					
AI biopsy GG	0	1	2	3	4	5
0	0	0	0	0	1	0
1	0	0	10	3	0	0
2	0	0	40	23	0	0
3	0	0	3	5	0	0
4	0	0	4	9	2	0
5	0	0	0	16	2	8
	RALP GG					
Pathologist biopsy GG	0	1	2	3	4	5
0	0	0	0	0	0	0
1	0	0	11	3	0	0
2	0	0	42	13	1	0
3	0	0	3	36	2	1
4	0	0	0	4	0	3
5	0	0	1	0	2	4

**Table 9:** Multi-regression models for adverse pathological findings at prostatectomy show a statistically significant correlation between the area of cribriform G4 and G5 for seminal vesicle invasion and lymph node metastasis, as G5 shows high OR for non-organ-confined disease.

Clinical Outcome	Tumor Area in mm^2^	Odds Ratio	95% CI	*p*
Non-organ-confined disease	G3	0.93	(0.78–1.07)	0.35
	G4	1.39	(0.99–2.04)	0.07
	Cribriform G4	1.89	(0.89–4.70)	0.12
	G5	48.52	(3.03–4125)	0.03 *
Seminal vesicle invasion	G3	0.82	(0.56–1.07)	0.22
	G4	0.72	(0.41–1.10)	0.17
	Cribriform G4	2.46	(1.16–5.45)	0.02 *
	G5	5.58	(1.57–30.56)	0.02 *
Lymph node metastasis	G3	0.73	(0.44–1.03)	0.13
	G4	0.8	(0.48–1.20)	0.32
	Cribriform G4	2.66	(1.23–6.06)	0.01 *
	G5	4.09	(1.25–20.12)	0.04 *

*: *p*-values < 0.05 were considered significant.

**Table 10:** Cox proportional-hazard regression analysis.

Parameter	Hazard Ratio	95% CI	*p*
Pathologist ISUP GG	2.09	(1.38–3.16)	<0.001 *
AI ISUP GG	1.80	(1.27–2.53)	<0.001 *
AI Gleason	2.15	(1.34–3.44)	<0.01 *
G3 area (mm^2^)	0.94	(0.77–1.14)	0.51
G4 area (mm^2^)	1.03	(0.99–1.06)	0.19
Crib G4 area (mm^2^)	1.13	(0.57–2.24)	0.72
Total G4 area (mm^2^)	1.03	(0.99–1.07)	0.18
G5 area (mm^2^)	1.10	(0.99–1.23)	0.07
Total cancer area (mm^2^)	1.02	(0.99–1.05)	0.22
G3 proportion of cancer (%)	0.13	(0.04–0.47)	<0.01 *
G4 proportion of cancer (%)	3.59	(0.65–19.72)	0.14
Crib G4 proportion of cancer (%)	2.94	(0.52–16.59)	0.22
Total G4 proportion of cancer (%)	3.97	(0.99–15.9)	0.05
G5 proportion of cancer (%)	243.7	(28.8–2062)	<0.001 *
**Strata**			
Pathologist ISUP GG1–2 versus 3–5	3.31	(1.19–9.23)	0.02 *
AI ISUP GG1–2 versus 3–5	5.91	(1.96–17.83)	<0.01 *

n = 126; *: *p*-values < 0.05 were considered significant.

## Data Availability

The patient identifiable data registry was used in this study based on the research permit and current legislation allowing the use of pseudonymized data for the research group. The metadata of the study cohort may be requested from the corresponding author. The precise AI model is the property of Aiforia Technologies Plc, and the queries on the model can be directed to Sami Blom, the second author in this report.
